# Another *de novo* mutation in the *SOD1* gene: the first Turkish patient with *SOD1*-His47Arg, a case report

**DOI:** 10.3389/fgene.2023.1208673

**Published:** 2023-08-25

**Authors:** Elif Bayraktar, Vildan Çiftçi, Hilmi Uysal, A. Nazlı Başak

**Affiliations:** ^1^ Suna and İnan Kıraç Foundation, Neurodegeneration Research Laboratory (NDAL), Research Center for Translational Medicine (KUTTAM), Koç University School of Medicine, Istanbul, Türkiye; ^2^ Department of Medical Biology and Genetics, Akdeniz University, Antalya, Türkiye; ^3^ Department of Neurology, Faculty of Medicine, Akdeniz University, Antalya, Türkiye

**Keywords:** fALS, sALS, SOD1, *de novo* mutation, His47Arg

## Abstract

Amyotrophic lateral sclerosis (ALS) is a fatal, progressive neurodegenerative disease of motor neurons. Most ALS cases are considered sporadic due to the presence of a combination of environmental and complex genetic risk factors, while approximately 10% of cases have a family history. Pathogenic variants in the *SOD1* gene are the second most frequent causative factor of genetics-based ALS worldwide, after *C9ORF72* hexanucleotide repeat expansion. The *De novo* occurrence of pathogenic mutations in ALS-associated genes and its effect on disease progression have been studied previously, especially in the *FUS* gene. Recent studies have shown that a very small portion of *SOD1* cases occurred *de novo*. Here, we present the first *de novo* case of the *SOD1* His47Arg mutation in a young female patient with mild symptoms and, currently, a slow progression for 7 years.

## Introduction

Amyotrophic lateral sclerosis (ALS) is a fatal, progressive neurologic disease characterized by the degeneration of upper and lower motor neurons from the motor cortex to the spinal cord. Symptoms include atrophy of the muscles, weakness, fasciculations, spasticity, dysarthria, dysphagia, and, sometimes, pseudobulbar involvement in the form of uncontrollable laughter or crying. Currently, ALS has no cure; thus, death usually occurs within 3–5 years from the onset of symptoms, mostly due to the degeneration of the respiratory muscles. Although 5%–10% of ALS patients consist of cases with a family history (fALS), the majority of ALS is sporadic (sALS) with a heritability of approximately 60% (Tang et al., 2019; Zhang et al., 2022). Genetic factors are the only causes confirmed to directly lead to disease development. Mutations in more than 50 genes are identified and associated with the pathogenesis of ALS; some of them also lead to frontotemporal dementia (FTD) (Wroe et al., 2008; Abramzon et al., 2020; Müller et al., 2022). The hexanucleotide repeat expansion in the first intron of the *C9ORF72* gene is responsible for most genetic cases in Europe (Renton et al., 2011). The first gene associated with ALS in 1993 was *SOD1*, the most common genetic cause among Asian patients and the second most common causative agent for familial cases after *C9ORF72* gene mutation in European populations (Zou et al., 2017). The other major genetic factors are two closely related DNA/RNA-binding proteins, TDP43 and FUS, which interfere with RNA processing mechanisms and cause proteinopathy when mutated (Van Deerlin et al., 2008; Kwiatkowski et al., 2009).

Genetic factors are not only responsible for familial cases (10%) but also for some isolated and apparently sporadic cases (Siddique and Siddique, 2021). Sporadic patients carrying an ALS-related mutation have been explained by 1) incomplete penetrance of the disease; 2) missing clinical information on the previous generations or they have not developed the disease; 3) misdiagnosis of the affected relatives or development of other phenotypes because of pleiotropy; 4) false paternity; or 5) occurrence of *de novo* mutations.

SOD1 is a highly abundant free radical scavenger enzyme that contains copper and zinc atoms. It protects the cells from oxidative damage caused by reactive oxygen species, specifically superoxide anions. Mutations in the *SOD1* gene cause a toxic gain of function (gof) through protein misfolding. Due to its gof nature, most of the mutations are inherited in an autosomal dominant manner. Only a small portion of *SOD1* mutations lead to disease development in both homozygous and heterozygous forms, the most famous such variant being the Asp91Ala change (Deng et al., 1993; Rosen et al., 1993; Andersen et al., 1995). More than 220 missense mutations have been identified in the *SOD1* gene since its discovery in 1993; however, only a quarter of them are proven to lead to ALS. This corresponds to 8%–23% in familial ALS cases and 1%–4% in sporadic or simplex cases in different populations (Wroe et al., 2008; Andersen and Al-Chalabi, 2011). In our Turkish cohort, published in 2020 based on 1,200 ALS patients, *SOD1* gene variants were detected in 13% of familial and 1.3% of sporadic ALS cases (Tunca et al., 2020).

Here, we report the first *de novo* case of *SOD1* c.140A>G p.His47Arg mutation among our updated number of 2,200 ALS cases. A 38-year-old Turkish woman with no family history and no parent consanguinity presented at the Akdeniz University Department of Neurology with early-onset and slowly progressing ALS. Whole-exome sequencing (WES) analysis was applied because of the atypical clinical features and young age of onset of the patient.

## Case description

**FIGURE 1:**
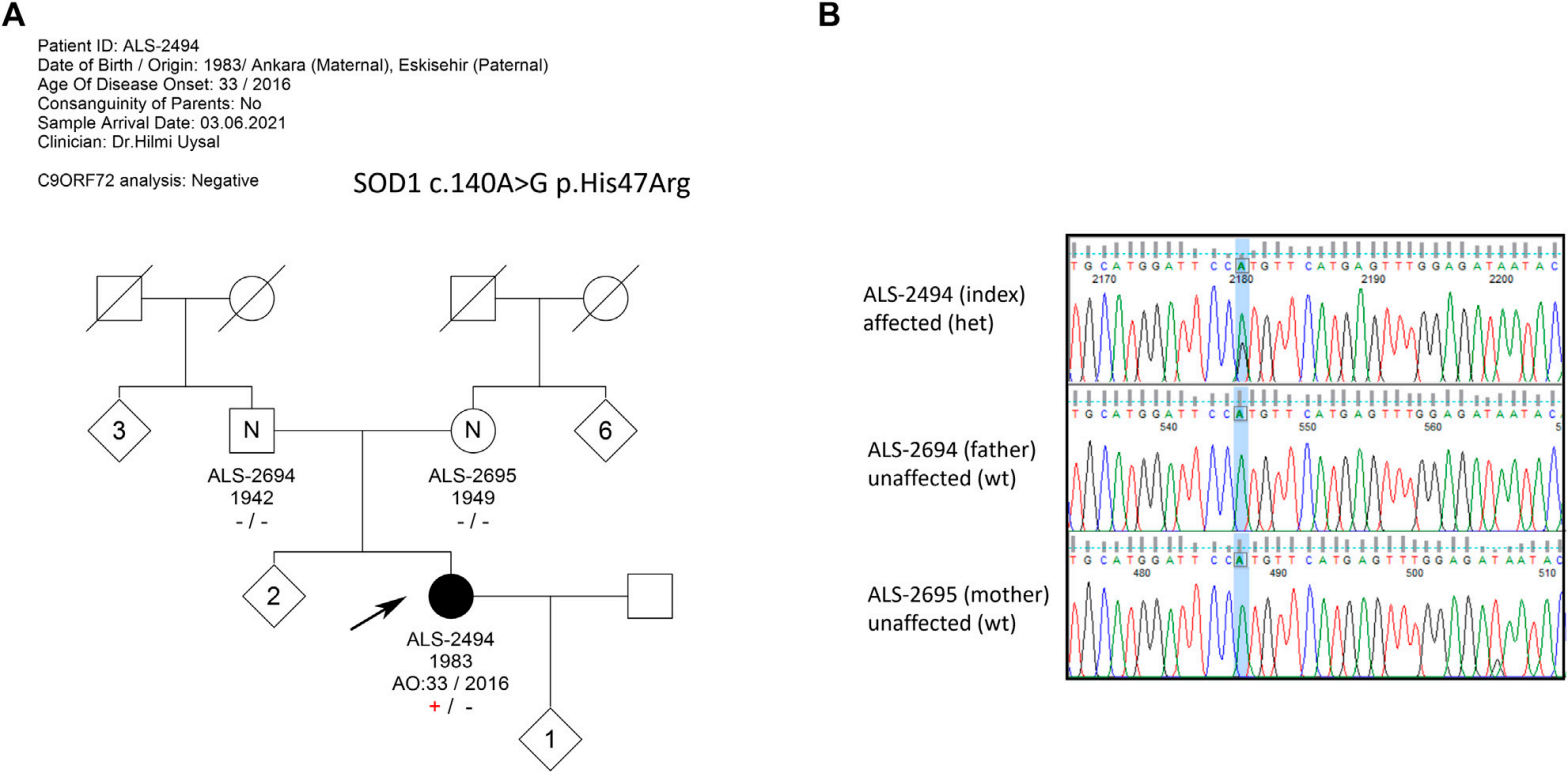
**(A)** Pedigree of the extended family with no ALS history; **(B)** Sanger sequencing results of family members. The index patient is heterozygous for c.140A>G mutation, while neither of the asymptomatic parents carries the mutation, confirming the *de novo* occurrence of the mutation.

The female patient, born 1983, first presented with a complaint of weakness in the right leg at the age of 33 in 2016. In the first 5 years, she had a slowly progressing disease; however, recently, weakness in walking and cramps became evident (2021). Although she does not have any symptoms in the hands, she feels infrequent fasciculations in her muscles. As of 2023, the patient is still alive. There are no similar complaints in the family and no previous disease history (Figure 1A).

In the examination of the patient, consciousness, speech, and high cognitive functions were all normal. No significant pathological finding was detected in the cranial nerve examination. Bulbar weakness was not detected. Jaw reflex was absent.

Upper extremity muscle strength was normal on the left distal and proximal muscles. On the right, there was no significant proximal weakness, while 4+/5 muscle strength and mild atrophy were detected in the distal first dorsal interosseous muscle and intrinsic hand muscles.

In the lower extremity, 2/5 muscle strength was present in the right distal foot dorsiflexion and plantar flexion. Distal foot muscles on the right were atrophic. There was 4+/5 muscle strength in the distal lower extremity on the left. Babinski reflex was positive on the right. Deep tendon reflexes (DTRs) were 3+ at the bottom. DTRs spread to the finger flexors on the upper right. Sensory examination revealed normal findings.

Cerebellar tests were within normal limits in the upper extremity. It could not be evaluated because of paresis in the lower extremity. However, it appeared normal.

No pathology was detected in the parameters examined in routine biochemistry and radiological investigation. Electromyography (EMG) showed that sensory action potentials in the upper and lower extremities were within normal limits. Although the motor conduction velocity study showed normal findings in the upper extremity muscles, compound muscle action potential (CMAP) could not be obtained from the extensor digitorum brevis (EDB) with bilateral peroneal nerve stimulation. In addition, CMAP could not be obtained from the abductor hallucis (AH) muscle with left tibial nerve stimulation, while a very low-amplitude motor response was obtained on the right.

Needle EMG revealed denervation findings in the distal and proximal muscles of the lower extremity, as well as the muscles of the right upper extremity and paraspinal thoracic muscles, and also neurogenic motor unit potential (MUP) changes and loss of MUP. Fasciculation was observed in the lower extremity muscles. In the transcranial cortical stimulation (TCCS) study, no motor evoked potential (MEP) response was obtained in the right lower extremity muscle. However, the MEP response could be obtained from tibialis anterior (TA) muscle with radicular stimulation. With all these findings, the patient was diagnosed with definitive ALS, according to the Revised El-Escorial Criteria, and riluzole was started in 2021.

The neurological examination of parents, both in their late ages, did not reveal the slightest subclinical ALS signs.

## Methods

The female index case with a clinical diagnosis of definitive ALS was referred to our laboratory for genetic analysis. The patient and her parents were informed and signed a written consent to join the genetic research. Their peripheral blood was collected in EDTA-containing tubes, and the genomic DNA was isolated using the MagNa Pure Compact System (Roche, Switzerland). The index sample was subjected to WES analysis after the patient tested negative for the *C9ORF72* hexanucleotide repeat expansion by repeat-primed PCR and fragment length analysis. Exome sequencing was conducted using an Illumina NovaSeq 6000 instrument at Macrogen Inc. (Macrogen, Korea). Raw data were uploaded to an online alignment, variant calling, and evaluation tool (SEQ platform by Genomize). Reads were mapped to the reference genome, and functional annotation was performed. Noncoding variants and synonymous variants other than those of splice regions were filtered out. Genes that are registered on the Online Mendelian Inheritance in Man (OMIM) database were selected, and all rare variants (MAF <0.01) were screened. The mutation was validated in the index case and family members with PCR and Sanger sequencing. The parental kinship was confirmed using the Promega PowerPlex 16 System (PPP16). PPP16 is a universal multiplex STR system for DNA typing. The kit, consisting of 15 highly polymorphic markers across the genome and the sex-specific amelogenin, is commonly and reliably used for parental genotyping, as well as for forensic analysis (Promega Corporation, Madison, WI).

## Results

WES analysis revealed the heterozygous presence of the *SOD1* c.140A>G p.His47Arg missense mutation in the index patient. In the WES analysis, no other variants with significant pathogenicity were detected in any of the genes associated with ALS and other neurodegenerative diseases. The variant was questioned in the patient and her parents by Sanger sequencing, and the heterozygous presence of the mutation c.140A>G in the index patient was confirmed. The parents, however, were found to be devoid of the aforementioned variant, pointing to a *de novo* occurrence of the mutation in the patient (Figure 1B). Blood samples of the two older sisters of the patient were not available.

## Discussion

The *SOD1*-His47Arg mutation was first described in two Japanese ALS families. It has been suggested that the substitution disrupted the active copper-binding region of the SOD1 protein, which reduces the enzymatic activity by about 80% in ALS patients compared to the unaffected family members. The cases from both families had a later age of onset and slower progression than other Japanese ALS families without *SOD1* gene mutations (Aoki et al., 1994). In 2002, it was reported that in another Japanese family carrying the His47Arg mutation, the disease had unique clinical features and slow progression compared to cases with the distal unilateral lower limb onset. The longest disease course of *SOD1* His47Arg-related ALS is recorded in this family, in a 71-year-old female subject with a survival of 47 years after the onset of the symptoms (Ohi et al., 2002). These studies suggested that this particular mutation might be associated with a new subtype of “*benign*” ALS with milder symptoms and much slower progression.

In an epidemiological study conducted in the Miyakonojo Basin region in southern Japan in 2001, three families with multiple affected individuals carrying the *SOD1* His47Arg mutation were investigated. Symptoms typically started in the lower extremities; the involvement of the upper extremities occurred in the following 2–15 years, and respiratory failure was observed after 6–30 years. In accordance with previous studies, it was determined that the lower extremity site-onset and slow progression were the characteristics of this mutation (Arisato et al., 2003). A patient of Pakistani origin was reported to have the *SOD1* His47Arg mutation, with lower limb onset and slow progression. The patient was diagnosed with familial ALS, identified in a family with similar clinical symptoms in the patient's mother, sister, and maternal uncle (Holmøy et al., 2007). Another family of Norwegian origin, diagnosed with Charcot–Marie–Tooth type 2 (CMT2) disease, was detected to have the complete penetrant His47Arg mutation with autosomal dominant inheritance. Patients were reported to encounter a preparetic phase, accompanied by muscle cramps and pain, lower extremity onset with predominant weakness, and atrophy, with unilateral and distal involvement. Since the patients did not meet the El-Escorial criteria of ALS, the mutation was also suggested to be a possible causative for phenotypes akin to hereditary motor neuropathy; therefore, patients with similar clinical findings should be screened for this mutation (Østern et al., 2012).

In 2019, a comprehensive study investigating *SOD1* mutations in 923 sALS and 159 fALS cases in a non-Caucasian ALS population was published (Tang et al., 2019). The His47Arg mutation was primarily reported in patients of Asian descent. Notably, it was the most frequently identified mutation in ALS patients of Chinese origin. The spinal-onset ALS patients carrying the His47Arg mutation had a mean age onset of approximately 50 years. They were reported to have slower progression and longer survival time than patients with other *SOD1* mutations. The findings were consistent with those of studies conducted in the European cohorts, strengthening the relationship established between the mild ALS/motor neuropathy phenotype and the *SOD1* His47Arg mutation (Tang et al., 2021). The clinical findings of *SOD1*-His47Arg patients described so far are given in Table 1. The Turkish patient reported here is highlighted in red.

**TABLE 1:** Clinical features of SOD1-His47Arg patients.

Family history	Origin	Mean AO (years)	Initial symptom	UMN inv.	Bulbar sign	N	Duration (years)	Reference
+	Japanese	49.6 (SD ± 10.9; *n* = 10)	LL	3 (*n* = 5)	1	13	17.3 (SD ± 10.7; *n* = 4)	Aoki et al. (1994)
+	Japanese	48.0 (SD ± 9.5; *n* = 14)	LL	1 (*n* = 1)	No	15	16.8 (SD ± 6.8; *n* = 9)	Aoki et al. (1994)
+	North American	43.2 (SD ± 11.7; *n* = 5)	n.a.	n.a.	n.a.	5	17.4 (SD ± 6.4; *n* = 5)	Juneja et al. (1997)
+	French	n.a.	LL	1 (*n* = 4)	No	4	11.5 (3–25 years; *n* = 4)	Camu et al. (1999)
+	Japanese	39.7 (SD ± 10.5; *n* = 9)	LL	3 (*n* = 9)	1	11	18.1 (SD ± 13.2; *n* = 9)	Ohi et al. (2002)
+	Japanese	44.3 (SD ± 8.7; *n* = 17)	LL	1 (*n* = 17)	1	17	12.1 (SD ± 7.6; *n* = 17)	Arisato et al. (2003)
+	Japanese	42.9 (SD ± 4.7; *n* = 7)	LL	No (*n* = 7)	No	15	17.2 (SD ± 8.1; *n* = 7)	Ohi et al. (2004)
+	Pakistani	55; (*n* = 1)	LL	1 (*n* = 1)	No	12	17–21 years; (*n* = 12)	Holmøy et al. (2007)
+	Japanese	50.3 (SD ± 4.18; *n* = 3)	LL	No (*n* = 3)	No	3	>38 (*n* = 1)*; >12 (*n* = 1)*	Yamashita et al. (2009)
+	Chinese	42.7 (SD ± 6.8; *n* = 4)	LL	1 (*n* = 4)	1	4	>12 (*n* = 4)*	Li et al. (2010)
+	Norwegian	46.7 (SD ± 9.4; *n* = 3)	LL	No (*n* = 3)	No	3	>5 (*n* = 2)*; ∼40–50 (*n* = 1)	Holmøy et al. (2011)
+	Chinese	54.5 (SD ± 11.1; *n* = 4)	LL	no (*n* = 4)	1	4	13 (*n* = 1) >3–12 (*n* = 3)*	Zhang et al. (2012)
+	Norwegian	42.5 (SD ± 10.9; *n* = 22)	LL	3 (*n* = 12)	1	22	29 (SD ± 13.3; *n* = 9)	Østern et al. (2012)
+/−	Chinese	51.4 (SD ± 6.9; *n* = 5)	LL	1 (*n* = 5)	No	5	>7–11 (*n* = 5)*	Zou et al. (2016)
+/−	Chinese	50.8 (SD ± 10.1; *n* = 8)	n.a.	n.a.	n.a.	8	>8 (SD ± 4.2; *n* = 3 + *n* = 5*)	Tang et al. (2019)
- (*de novo*)	Turkish	33; (*n* = 1)	LL	1	No	1	>7 (*n* = 1)*	Our study

Family history: + represents inherited disease; +/- represents the studies where both familial and apparently sporadic cases were reported; AO, age of disease onset, represented as the mean ± standard deviation; n, number of affected cases included in the data; initial symptom: LL, lower limb predominance; UMN, inv: number of cases showing upper motor neuron involvement in addition to lower motor neuron findings; n, number of affected cases studied; bulbar signs, number of patients showing bulbar involvement; N, number of affected members in the study cohort; duration, time between the symptom onset and death. Data are presented as the mean ± standard deviation or range of years. *n*, number of affected patients included in the data. *, still alive at the time of the study. n.a, information not available.

The genetic alterations observed for the first time in a family are called *de novo* mutations. They may occur in parental gonadal cells or in early and late postzygotic embryonic developmental processes. Mutations that occurred in the parental germ cells and the early zygote are observed in all three embryological layers in the offspring and are expected to be observed in all tissues in the next generations, explaining how the heterozygous dominant *de novo* mutations may be observed in affected offspring but not in parents (Al-Chalabi et al., 2014; Nicolas and Veltman, 2019). The occurrence of a postzygotic mutation in the ectoderm layer might only affect the tissue that forms the nervous system; therefore, the variant will not be further inherited, and it can also not be detected in blood-derived DNA. The importance of obtaining postmortem samples from affected tissues in apparently sporadic ALS patients becomes more prominent with these studies.

**FIGURE 2:**
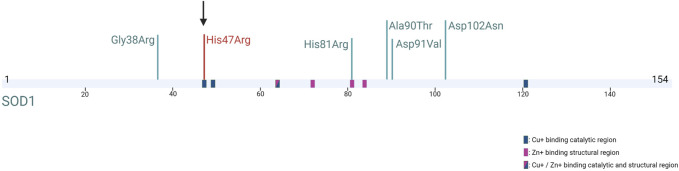
SOD1 protein structure and five previously reported *SOD1 de novo* mutations (in green) (Müller et al., 2022; Alexander et al., 2002) and our mutation (in red) (Created with BioRender.com).

Recently, the *de novo* occurrence of *SOD1* mutations was investigated in a large cohort of ALS patients, and it was confirmed that five isolated cases had pathogenic *de novo* alterations in the *SOD1* gene, the first one being the “true sporadic” case described in 2002 by Alexander et al. (2002). The symptoms were similar to those of the inherited *SOD1*-related ALS disease of the same variant, and all these variants were described in familial form, in different populations (Müller et al., 2022). In our patient, the absence of a notable difference in clinical findings compared to the inherited form of His47Arg is coherent with the other *de novo SOD1* cases described in the literature (Figure 2). The earlier disease onset observed in most patients with a *de novo SOD1* mutation might be attributed to the genetic and environmental modifiers, as well as the origin of the mutation.

Given that the *de novo* occurrence is observed mostly on previously reported residues, some codons on the *SOD1* gene are believed to be prone to mutations (hotspots); thus, the tendency to *de novo* pathogenesis in the *SOD1* gene also contributes to the increase in the *SOD1*-related ALS frequency worldwide. Similar to the other *de novo SOD1* mutations reported, the Turkish His47Arg mutation is also a variant previously described in a familial context in other populations. A comprehensive haplotype analysis study for the most common ALS mutations, such as Ala5Thr, His47Arg, Asp91Ala, Leu118Val, and Leu145Phe, would be informative in detecting the *de novo* frequency on these residues.

Here, we report, for the first time, a Turkish patient with a *SOD1-*His47Arg mutation; this is the first report of a *de novo* occurrence of His47Arg in the *SOD1* gene, in which only five *de novo* mutations have been described so far. Our study may shed light on the complex pathogenesis of *SOD1*-based disease and also on *de novo* mutations in *SOD1* and in other ALS genes, which may be more crucial in the pathogenesis of ALS than previously recognized.

## Data Availability

The data presented in the study are deposited in the European Nucleotide Archive (ENA) repository, accession number PRJEB64663.

## Abbreviations

AH, abductor hallucis; ALS, amyotrophic lateral sclerosis; C9ORF72, chromosome 9 open reading frame 72; CMAP, compound muscle action potential; CMT2, Charcot–Marie–Tooth type 2; DTR, deep tendon reflex; EDB, extensor digitorum brevis; EDTA, ethylenediaminetetraacetic acid; EMG, electromyography; fALS, familial ALS; FTD, frontotemporal dementia; FUS, fused in sarcoma; gof, gain of function; MAF, minor allele frequency; MEP, motor evoked potential; M-Response, motor response; MUP, motor unit potential; OMIM, Online Mendelian Inheritance in Man; PCR, polymerase chain reaction; sALS, sporadic ALS; SOD1, superoxide dismutase 1; TA, tibialis anterior; TCCS, transcranial cortical stimulation; TDP43, TAR DNA-binding protein 43; WES, whole-exome sequencing.
